# Quality Coding by Neural Populations in the Early Olfactory Pathway: Analysis Using Information Theory and Lessons for Artificial Olfactory Systems

**DOI:** 10.1371/journal.pone.0037809

**Published:** 2012-06-18

**Authors:** Jordi Fonollosa, Agustin Gutierrez-Galvez, Santiago Marco

**Affiliations:** 1 Department of Electronics, Universitat de Barcelona, Barcelona, Spain; 2 Artificial Olfaction Group, Institute for Bioengineering of Catalonia, Barcelona, Spain; Monell Chemical Senses Center, United States of America

## Abstract

In this article, we analyze the ability of the early olfactory system to detect and discriminate different odors by means of information theory measurements applied to olfactory bulb activity images. We have studied the role that the diversity and number of receptor neuron types play in encoding chemical information. Our results show that the olfactory receptors of the biological system are low correlated and present good coverage of the input space. The coding capacity of ensembles of olfactory receptors with the same receptive range is maximized when the receptors cover half of the odor input space - a configuration that corresponds to receptors that are not particularly selective. However, the ensemble’s performance slightly increases when mixing uncorrelated receptors of different receptive ranges. Our results confirm that the low correlation between sensors could be more significant than the sensor selectivity for general purpose chemo-sensory systems, whether these are biological or biomimetic.

## Introduction

Animals’ sense of smell has been shaped over evolutionary time to perceive the environment and extract essential information for their survival. It provides relevant information to locate food, detect potential dangers such as predators or rotten food, and to mediate in reproductive behavior. The sense of smell is a wonderful general purpose chemical sensing system. For certain figures of merit such as specificity, response time, detection limit, coding capacity, time stability, robustness, size, power consumption and portability, it clearly outperforms analytical chemical instrumentation. Electronic noses appeared in the early 90s as smart chemical sensing instruments with an architecture inspired by the olfactory pathway [1]. However, insufficient understanding of the chemical information coding and information processing in the biological system means that biomimetics are reduced to a superficial level. It has been argued that an increased level of bioinspiration in the design of these instruments could lead to new paths of innovation [2].

The olfactory system is thought to be adapted to the statistical properties of the set of chemicals to which it is exposed. The “efficient-coding hypothesis” [3] has been explored by Kostal et al. [4] focusing on intensity coding with the use of information theory techniques. However, the analysis of quality coding, i.e. the early olfactory system’s ability to detect and discriminate different odors, in an information theory framework has received less attention. In this paper, we explore the performance of the olfactory system in identifying the quality of the input stimuli and we use the term coding capacity to quantify the number of odorants that can be coded with a set of receptors.

The input stage of the olfactory system consists of a complex arrangement of Olfactory Receptor Neurons (ORNs) distributed over the nasal epithelium to detect airborne chemicals. Individual ORNs express a single type of Odorant Receptor (OR) [5]–[6]. The number of types of OR depends on the species and varies from a few tens in insects to several hundreds in vertebrates (e.g. 387 types of functional ORs in humans and 1,035 types in mice. [7]–[8]).

There is a large body of evidence based on electrophysiology that has consistently indicated that each olfactory neuron can respond to a variety of odorants and each odorant can bind to different receptors [9]. Since different neurons respond to a different set of odorants, this establishes the principle of the combinatorial code [10]. This principle also applies to other biological senses where a large set of stimuli must be discriminated by a set of different receptors. In particular, the coding capacity problem in the senses of vision [11]–[12] and taste [13]–[14] has also attracted the interest of the scientific community.

Even though statistical techniques have been applied to predict the specificity of OR [15], the distribution of OR selectivities in mammals still remains unclear. ORs can be specific and respond selectively to one single steroid [16] or detect odorants that share particular physicochemical properties such as molecular size or structure [17]. In fact, broadly tuned ORs have also been identified [18]–[19]. Moreover, receptors have been reported to be very specific for certain molecular features, but very unspecific for others. [20]. Therefore, each odorant seems to bind with a collection of ORs of different specificity levels and the combined response of the ORs mediates in odor identification.

Experimental analyses focusing on the study of the specificity of olfactory receptors have been presented in the past. Duchamp-Viret et al. used sixteen pure odor compounds as stimuli in rats and the response of ninety ORNs was recorded. The Receptive Range (RR) according to a 6-odor subset showed a broad distribution of receptors [21]. Hamana et al. isolated 2,740 mouse receptor neurons and studied their specificity to a chiral pair of odorants showing that more than 80% of the responsive receptors have sensitivity overlap [22]. Araneda et al. stimulated different octanal receptors with nine odorants to reveal that some receptors have broad RR while others were activated only by octanal [23]. Soucy et al. studied the similarity of receptors, which directly measures the overlap between the RRs of different receptors, to show that nearby glomeruli tend to have very diverse odor sensitivities in rats and mice [24]. In a very comprehensive study, Hallem measured individual olfactory receptors of the drosophila antennae with a panel of over 100 odors. The results showed that *Dmelanogaster* receptors range continuously from narrowly tuned to broadly tuned [25].

However, to the best of our knowledge, no classification of rats’ olfactory receptors according to their RRs when exposing them to a large number of odorants has been reported before. The large number of receptors and more significantly, the immense number of potential ligands, require a titanic experimental effort to comprehensively characterize and understand the interactions between odorants and receptors [26].

Individual ORNs expressing the same type of receptor converge in a very orderly fashion into one or two glomeruli of the Olfactory Bulb (OB) [5]–[6]. Therefore, each odorant produces a specific glomerular activity pattern [27]. As a consequence, the chemical properties captured by the ORNs can be seen as a 2-dimensional activity map at the glomerular level. Current trends in olfactory research indicate that chemical information is captured not only following a spatial code but also a temporal code [28]. At present, the division of information content between the spatial activation map and the temporal firing patterns remains unknown.

The role of the RR in different populations of ORNs has been studied theoretically for odor coding analysis. Sánchez-Montañés et al. applied multi-component chemical stimuli to a simple and linear model of the sensory neuron response. The receptive field was characterized by a pattern of sensitivities and they used information theory tools to demonstrate that OR populations with broadly tuned receptors perform better in estimations than perfectly specific receptors [29].

Alkasab et al. presented a very simple and straightforward approach for modeling the complete OR population [30]. They explored how the information coding capacity of the system is directly affected by the RR of the OR. The model is based on a three-dimensional abstract finite space that represents the odor space. In this model, every point in this space represents a different odor and every receptor is represented by a cube. Receptors provide significant response if the point representing the odorant falls within the cube, whereas if the point falls outside the cube the receptor is not responsive. So, receptors are binary entities and the size of the cube represents the RR of the receptor. In this simple model, every odor is characterized by only three molecular descriptors (three-dimensional cube). The real dimensionality of the input space is unknown and thousands of molecular descriptors can be used to classify odorants. In any case, there is consistent agreement that the dimensionality of this odorant space has to be very large. Alkasab et al. distributed the input odorants (stimuli) according to a uniform random distribution in the odor space. Then, using Information Theory tools, they quantitatively studied the capacity of the system to code the input stimuli depending on the number, position and sizes of the cubes that model the receptors [31].

The olfactory system is an appealing model for inspiration when creating general purpose artificial chemical sensor systems due to the excellent coding efficiency and the large number of odorants that can be detected and discriminated [32]–[33]. This partially motivates the work presented in this paper, where we analyze the encoding of chemical information in the first stages of the olfactory system. In particular, the aim of this paper is to study how the coding capacity depends on the distribution of specificities, the RR and the correlation among receptors. This is inferred from the activity of a rat’s olfactory bulb when exposed to a large number of odorants.

In this paper, instead of relying on simplified theoretical models, we have applied Alkasab et al. approach to actual data from biology. We have analyzed a dataset of glomerular activation in rats across a large set of odorants to plot the distribution of specificities of the ORs. We studied the different tuning of the olfactory receptors and its contribution to the outstanding performance of biological olfaction in terms of coding capacity. We have quantitatively analyzed the odor coding capacity of different sized ensembles and different types of receptors and compared their performance to the theoretical model presented by Alkasab et al. It is our belief that a better understanding of odor coding in olfaction may provide valuable insights for the design of general purpose Artificial Olfaction Systems.

## Materials and Methods

### Glomerular Activity Maps Dataset

To perform this study, we used the OB activity dataset compiled by the group of Leon & Johnson at the University of California in Irvine [34]–[36] and made publicly available through the Glomerular Activity Response Archive website at http://gara.bio.uci.edu. The activity across the entire glomerular layer of the rat OB was systematically mapped using uptake of [^14^C]-radiolabeled 2-deoxyglucose (2DG). They captured OB activity in response to a large set of odorants with different chemical structures. We would like to emphasize that mapping the entire glomerular layer, in contrast to other techniques, is a particularly interesting facet of this dataset. On the other hand, this technique fails to capture the temporal information.

The two-dimensional activity map (44x80 pixels) is obtained after blank subtraction and the data across rats exposed to the same stimulus was averaged to obtain the activity maps. To test the variability across individuals exposed to the same odorant, indices of pattern dissimilarity were calculated with the data resulting from 35 rats exposed to different stimuli [37]. The pairs of rats exposed to the same odorant showed lower pattern dissimilarity values compared to the pairs of rats exposed to different stimuli. The difference between same-odorant and different-odorant pairs was tested under a Mann-Whitney U-test and it showed that the difference is statically significant (U = 12240, P<0.0001).

Compounds that show constant odor quality in humans with stimulus concentration were systematically exposed to rats at different concentrations. The olfactory bulb was mapped and the activity patterns evoked were constant when expressed in units of z-scores. However, odorants that show different odor quality in humans for different stimulus concentration gave different activity patterns when exposed to rats at different concentrations [38]. Units in each data matrix were, therefore, normalized to z-scores relative to the mean response and the standard deviation of values across that matrix to eliminate the dependence of the OB activity with the odorant concentration. That is, by using z-scores the analysis focuses on odor quality coding, while rejecting variations due to odorant concentration.

Therefore, the Leon & Johnson dataset is suitable for statistical analyses and study of the OB activity evoked by different odorants. However, due to the limitations of the imaging technique, the present study disregards the time coding and only considers the spatial component of the response code.

The complete dataset includes 472 group-averaged activity maps in response to 339 different odorants, some of them at different concentrations, with some replications of the same exposure conditions. However, repetitions of the same odorant in the dataset could bias our conclusions. Therefore, for each chemical we limited the activity maps to the lowest concentration measurement for a total analysis of 339 different patterns.

Sectioning perfectly is a challenge, particularly in the ventro-caudal and dorsal parts of the OB, and minor tissue damage may occur. Therefore, as a consequence of the experimental procedure, most of the activity maps contain missing values, mainly distributed in the ventro-caudal and dorsal parts and on the border of the activity map. In order to have the same odor input space for all the receptors, we considered only the pixels that were not damaged in any of the activity maps measured and thus show significant response (positive or negative) to all the odorants. The result is that we obtained maps of 1,778 active pixels for 339 different odorants. In addition, due to difficulties related to image alignment, the variance in mounting the tissue prior to sectioning and the different size and shape of each rat OB, a minor uncertainty in pixel position has to be taken into account in subsequent discussions. In our analysis, since no chemotopic order is apparent and distant and nearby glomeruli show the same odor sensitivity variability [24], every pixel in the image is considered as if it were a chemical receptor type.

To use the Alkasab et al. methodology approach based on binary receptors, a pixel was considered as a “positive response” to one odorant if its value is positive and a “null response” if the pixel response is negative. In this work, we define the RR of the receptors as the ratio between the number of analytes at which the receptor shows a “positive response” and the total number of odorants (339). Please take into account that further discussions concerning the role of the RR in coding capacity are dependent on the definition used in this work.

### Calculation of Mutual Information

We studied the capabilities of different subsets of receptors to capture information about the stimuli presented. We created different combinations of receptors, chosen according to their RR, and estimated the capacity of the subset to encode the odorants of our database.

The main information measurement is entropy. This quantifies the difficulty in predicting the state of a system with no other information. The average entropy *S* can be expressed as:
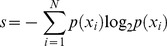
(1)where *N* states are possible and *p(x_i_)* is the probability of presenting state *x_i_*. When the log is taken to base two, the entropy is in units of bits.

On the other hand, Mutual Information (*MI*) determines the information of a random variable contained in another related random variable and quantifies how the uncertainty of the first variable is reduced when the state of the second variable is known. The *MI* of two completely independent variables is zero and, at the other extreme, if the variables are identical *MI* equals the entropy. For two discrete random variables *X* and *Y*, *MI* can be expressed as [39]:
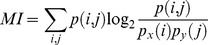
(2)where *p_x_(i)* and *p_y_(j)* are the marginal probability distribution functions of variables *X* and *Y* and *p(i,j)* is the joint probability distribution function. For discrete variables (or continuous variables after a quantization step), *MI* can be estimated from the histogram approximation of the probability density function, that is, by counting the number of points falling into the various bins. Hence, *MI* is determined by simply counting the points falling into the *i*th bin of *X*, into the *j*th bin of *Y*, and into their intersection [40].

In the study of odor coding and with the hypothesis of equal priors for the different odorants, the entropy is *S* = log_ 2_ *N*; where *N* is the number of possible stimuli (odorants) presented to the system (subset of receptors). So, MI quantifies the information about the stimulus X (odor quality) given by the state of the receptors Y (binary response word). Therefore, MI measures the ability of the complete set of receptors to make discriminations over repeated stimulus applications and quantifies the uncertainty reduction of guessing the stimulus presented. The MI has, therefore, a direct relationship with the number of stimuli that can be coded by the set of receptors (coding capacity) since it is possible to code 2^MI^ different odors. When MI reaches S, the response of the ensemble of receptors is able to perfectly encode the presence of any individual stimulus in the system.

Our dataset comprises 339 different odorants (possible stimuli). However, for the sake of simplicity and clarity, the MI was calculated with random subsampling using sets of 256 different stimuli, which limits the maximum performance of the receptor array to 8 bits. We assumed that all the odorants appear with the same frequency in nature and, therefore, we selected the stimuli according to a uniform distribution.

**Figure 1:**
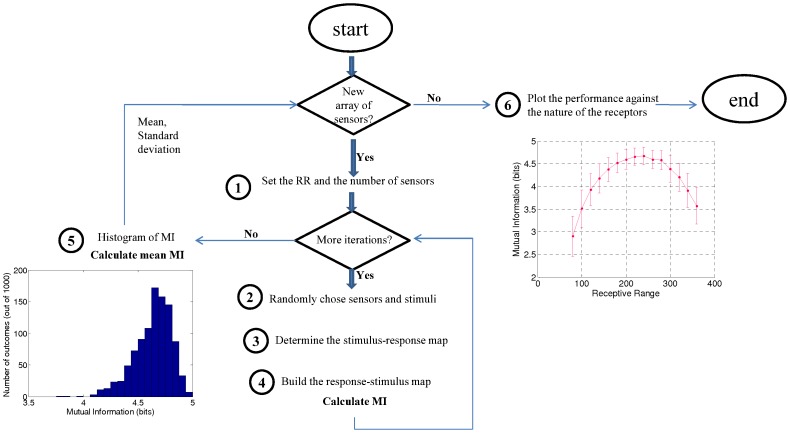
Method to estimate the coding capacity of groups of receptors. This is the routine to calculate the MI for different receptor type ensembles. In step 1 we set the number and the RR of receptors. In step 2 we randomly select the stimuli and receptors from the database. In step 3, the stimulus-response map is calculated, and reversed in step 4 to calculate MI. We select a new set of receptors (of the same type) and stimuli and repeat the cycle thousands of times. We obtain the histogram of MI and we calculate the mean value and the standard deviation of the values obtained for the same type of receptors (step 5). Then, we change the number and the RR of receptors (step 1) and compute the routine again. Finally, in step 6, we plot the performance of the ensemble across the type of receptors.

Figure 1 shows the routine used to calculate the MI, which is analogous to the method proposed by Alkasab et al [30]. Firstly, we set the number and the mean RR of the receptors. In the second step, the receptors and 256 stimuli are randomly chosen from our database. For the receptors, minor variations around the target RR are permitted to avoid repetitions (the receptors were randomly chosen from those that show “positive response” to the same number of odorants ±3). In step 3, the binary response of the ensemble of receptors to the stimuli is evaluated to determine the stimulus-response map. At this point we obtain a table with 256 binary words, each of them representing the binary response of a receptor to the 256 stimuli. The table is reversed in step 4 to obtain the response-stimulus map, that is, the response for a stimulus across receptors. We list the codes evoked with the associated stimuli to calculate the MI. Then, a new set of receptors and stimuli are chosen and the cycle is repeated one thousand times. In step 5, we obtain the histogram of MI and we calculate the mean value and the standard deviation. Finally, we change the type and/or the number of receptors and compute the routine again (back to step 1). In step 6, we plot the performance of the ensemble of receptors against the receptor type.

## Results

### Receptive Range Distribution of the Receptor Type Population

**Figure 2:**
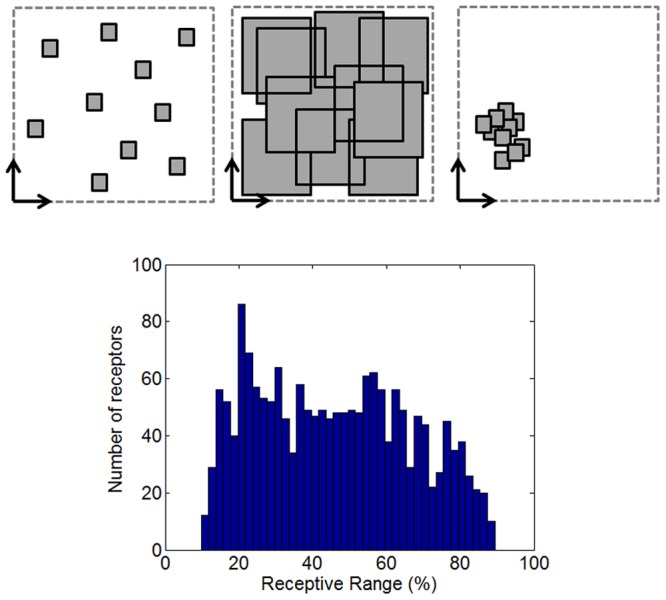
Interrelationship among receptive range, selectivity and correlation of olfactory receptors. Top: **Selectivity and correlation of olfactory receptors.** The odor space and the RR of the receptors are represented by the dashed-square and the black squares respectively. Two different 10-receptor arrays are created: with narrowly (left) and broadly (center) tuned receptors. Narrowly tuned receptors may be less correlated, while broadly tuned receptors cover a larger area of the odor space and respond to a larger number of odorants. While broad RR receptors could be more correlated (more overlap between receptors), receptors with small RR may also be correlated (right). Bottom: **Receptive range distribution.** Receptive range (RR) distribution for the 1,778 active receptors. More selective receptors respond to a lower number of odorants (low RR) and broadly tuned receptors show a “positive response” to most of the odorants (high RR). Total of different odorants tested: 339.

The RR directly determines the selectivity of the receptors. On the one hand, very narrowly-tuned receptors show a positive response to a low number of odors and their specificity is high. On the other hand, broadly-shaped receptors cover a larger area of the odor space and show a response to a significant number of odors. However, as the receptors’ RR increases, the potential overlap between receptors is higher and the receptors could have greater correlation. Figure 2 (top) shows the trade-off between the selectivity and the coverage of the odor space for narrowly-tuned receptors (left) and the sensor correlation for very unspecific receptors (center) for a 10-receptor array. However, eventually we may have narrowly-tuned receptors that remain correlated (right).

**Figure 3:**
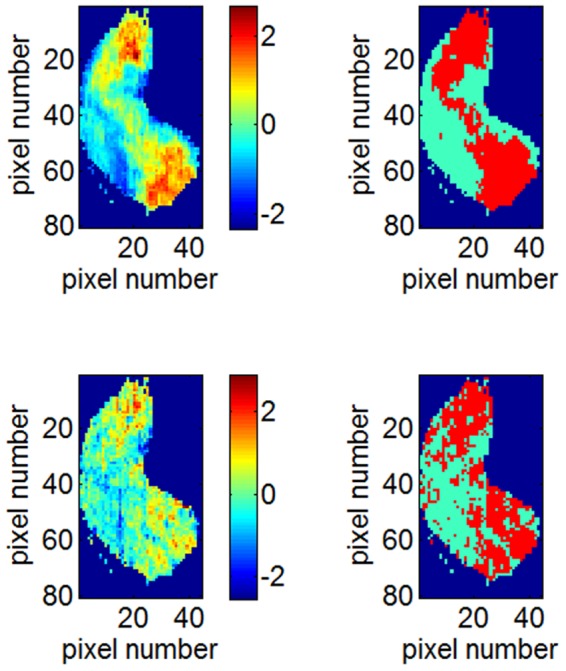
Olfactory bulb activity images. The olfactory bulb activity measured gives a pattern obtained using uptake of [^14^C]-2DG when exposed to 2-ethylfuran (up, left) and exposed to 1,7-octadiene (bottom, left). The corresponding binary map of the olfactory bulb activity for 2-ethylfuran (top, right) and for 1,7-octadiene (bottom, right). Red: “positive response”, sky-blue: “null response”, dark-blue: background.

As explained in the Methods section, a pixel is considered to respond to one odorant if its activity is over the mean activity across the image for this particular chemical. Figure 3 (left) shows the activity maps obtained for 2-ethylfuran (top) and 1,7-octadiene (bottom) and illustrates the variability of the glomerular activity for these odorants. Figure 3 (right) shows the corresponding binary activity.

We analyzed the selectivity of the chemical receptor population with the activity maps for 339 different odorants and sorted the receptor types (pixels) according to their RR. When considering all odorants, the most selective receptors show a “positive response” to only a few species (to 40 different odorants, i.e. 10% of the RR). It is quite possible that there are ORNs more selective than this, but our results are probably limited by the experimental technique (the reader should take into account that every single image corresponds to an average image over a few animals).

At the other extreme, broadly-tuned receptor types exhibit a “positive response” for most of the odorants (up to 90% of the RR). Figure 2 (bottom) shows a histogram of the RR (related to the selectivity of the receptor type) for the 1,778 receptors when exposed to all the odorants. From this figure we can conclude that the ORN distribution continuously covers a wide range of RR, from very selective to very unspecific ORN. This distribution is consistent with the pattern encountered in previous studies for the drosophila [25], measuring a panel of over 100 odors and in rats using few odorants [21] and it suggests that very different species share a broad RR distribution.

### Coding Capacity of Homogenous Receptor Type Ensembles

We selected different groups of *n* receptors with similar RR and explored the corresponding coding capacity when changing the RR from 12% to 88%, and when increasing the number of receptors.

As described in the methods section, for each ensemble the receptors were randomly chosen with minor variations in the RR. We calculated the MI of these receptor-type ensembles with sets of 256 stimuli chosen according to a uniform distribution. Consequently, the entropy of the discrimination task is 8 bits.

**Figure 4:**
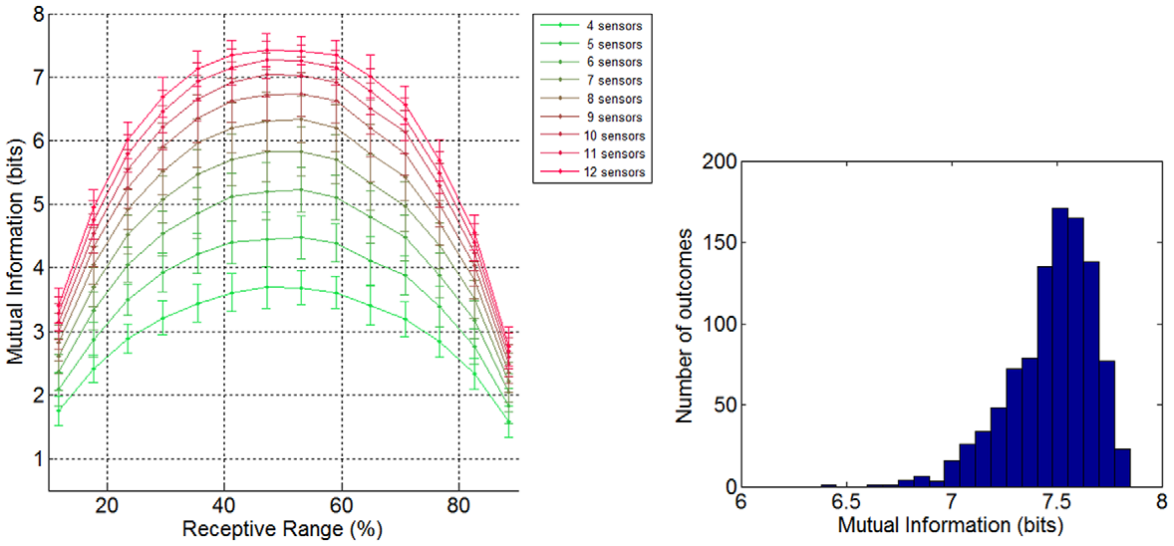
Mutual Information for odor coding. Top: **Mean performance of different sized arrays of receptors and different receptive range.** Mean and standard deviation (after 1,000 repetitions) of the evaluated coding capacity for homogenous groups of 4, 5, 6, 7, 8, 9, 10, 11, and 12 receptors for different RR of the receptors. The MI was calculated with sets of 256 stimuli, which limit the maximum array performance to 8 bits. The coding capacity increases with the number of receptors and there is an optimum coding capacity when the RR is about 50%. More selective (less RR) or less selective (more RR) gives a degraded performance. Bottom. **Performance distribution of a 12-receptor array.** Distribution of calculated MI for a 12-receptor array and 53% RR, after 1,000 trials. The histogram corresponds to step 5 of the routine (see figure 1) and is used to calculate the mean performance and standard deviation of the ensemble (top).

Figure 4 (left) shows the mean performance for different sizes (4, 5, 6, 7, 8, 9, 10, 11, and 12 receptors) and different RR (from 12% to 88%) of homogenous receptor-type ensembles, after 1,000 random subsampling cycles for each ensemble cardinality. Figure 4 (right) shows the performance distribution of a 12 receptor-type ensemble.

**Figure 5:**
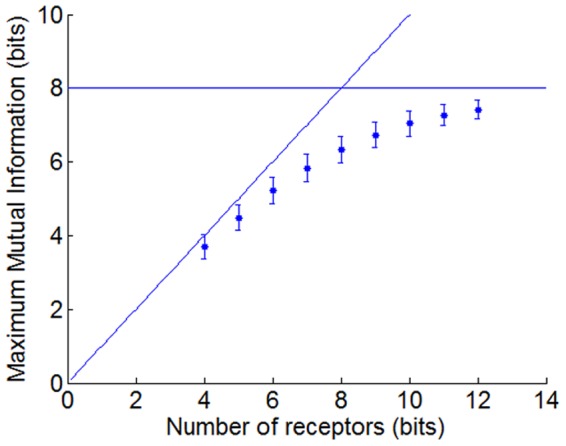
Maximum Mutual Information across a number of receptors. The coding capacity increases for larger ensembles of receptors. However, the MI is bound by the maximum entropy of the discrimination task, in this case 256 stimuli (8 bits).

Several conclusions can be drawn from figure 4. On the one hand, it shows that the coding capacity increases with the number of receptors, even if the incremental gains become smaller the bigger the sensor array is (see figure 5). On the other hand, it is clear that there is an optimum coding capacity when the RR is about 50%. More selective (less RR) or less selective (more RR) receptor types show a degraded coding capacity. Obviously, at the extreme of highly selective receptors, the ability to code for odors is equal to the number of receptors, while for perfectly correlated sensors they can only code a single odor.

It may be argued that this result is related to the fact that the number of codes for a certain receptor set is maximal when half of the receptors are active:
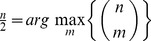
(3)However, this argument looks at codes across pixels, whereas the receptor range measures the code across the odorants. We could envision a situation whereby codes have 50% of the binary digits set to 1, but these receptors are extremely correlated. In this situation, the coding capacity of the array would be minimal although the number of potential codes would be very high.

In the theoretical model presented by Alkasab et al. [30], the optimum RR is shifted to smaller values, especially for arrays with many receptors (optimum RR = 25% for 128 sensors). From our analysis, this may be a model artifact due to an imposed restriction in the location of the three-dimensional cube that models the RR, which must be completely inside the finite volume considered (odor space). As the cube (RR) becomes larger, the probability of containing the region at the centre increases. The result is that if the stimuli fall in a central region, they activate all the receptors and the stimuli become undistinguishable. Therefore, the coding capacity of the modeled sensor array starts to drop before RR = 50%.

Figure 5 shows the maximum homogenous ensemble performance of different sized ensembles. Adding more receptors to the array increases the performance of the ensemble since the new receptor types can cover new areas of the olfactory space. However, as the number of receptors in the array increases, the addition of more receptors contributes less to the ensemble performance because there is an upper boundary given by the difficulty of the discrimination task set at 8 bits (256 stimuli).

### Sensor Diversity: Coding Capacity of Heterogeneous Receptor Type Ensembles

Figure 2 (bottom) shows that the selectivity of the olfactory neurons covers from RR = 10% to RR = 90%. In this section, we report results concerning the odor coding capabilities of heterogeneous receptor-type arrays compared to homogenous receptor-type ensembles.

**Figure 6:**
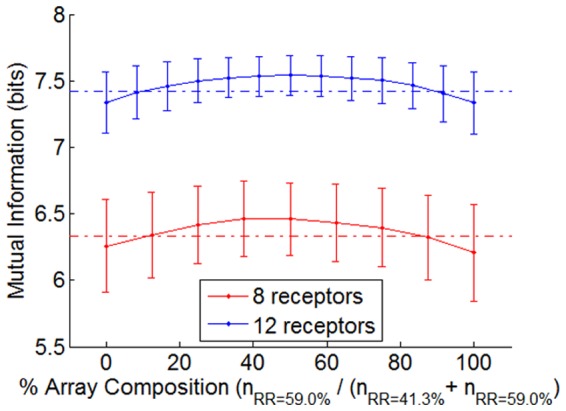
Array performance of heterogeneous ensembles. Mean and standard deviation (after 1,000 repetitions) when mixing receptors with RR = 59.0% and receptors with RR = 41.3%. Dashed horizontal lines show the maximum performance for 8 and 12 receptors when limited to homogenous arrays. Heterogeneous mixtures perform 0.15 bits better than homogenous arrays.

Heterogeneous ensembles of 8 and 12 receptors were made by mixing receptors of RR = 41.3% and RR = 59.0%. In Figure 6 the performance of these arrays across the degree of mixing when exposed to 256 stimuli are presented.

We found a heterogeneous mixture of types of receptors (half of the receptors with RR = 41.3% and the other half with RR = 59.0%) that performs at least 0.15 bits better than the best configuration of homogenous ensembles (all the receptors with the same RR of about 50%; see figure 4, left).

We can conclude that increasing the diversity of RR in the ensembles of receptors improves the coding capacities of the biological olfactory system, but only with a very minor incremental gain. The increase that we found by using heterogeneous ensembles is less than the improvement presented by Alkasab et al. in his theoretical model [30]. This higher increase in the theoretical model can be explained by exploring the correlation between receptors.

### Receptor Type Correlation

Correlation among receptor types could have a clear impact on the coding capacity of the receptor ensemble. On the one hand, correlation can increase redundancy and noise robustness, but on the other hand, the coding efficiency will probably be reduced. See figure 2 for a better understanding.

**Figure 7:**
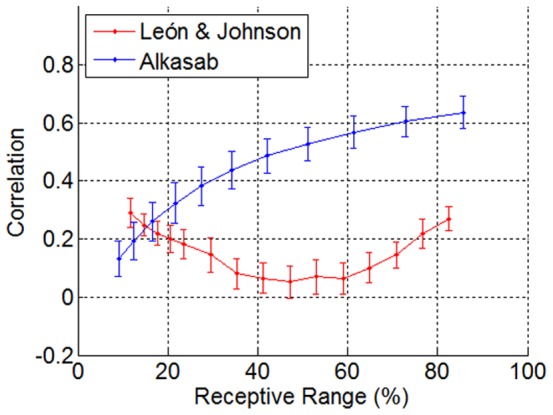
Correlation between pairs of sensors of similar receptive range. Mean correlation (after 2,000 repetitions) between pairs of sensors of similar RR, for the theoretical model of Alkasab et al. (blue) and for the measured data across the rat olfactory bulb (red). Biological data show low correlation values (always below 0.4).

Figure 7 shows the Pearson correlation for pairs of receptor types exposed to 256 odorants, for both the Alkasab et al. [30] model and the measured glomerular activity data.

The most interesting conclusion from figure 7 is that biological data shows low correlation values (always below 0.4). Although the methodology is slightly different, these results are in agreement with the results reported by Soucy et al. [24], whose results show mean correlation values for glomeruli below 0.3.

The maximum performance of homogenous arrays is RR = 25–30% for the Alkasab et al. model and RR = 47–53% for biological data, which corresponds to a correlation of ρ = 0.35 and ρ = 0.06 respectively. Alkasab et al. made heterogeneous ensembles by mixing receptors with RR = 10–15% (ρ = 0.2) and RR = 50–55% (ρ = 0.55). However, we mixed receptors with RR = 41.3% (ρ = 0.07) and RR = 59.3% (ρ = 0.06), which are scarcely correlated. Hence, it is difficult to find sets of heterogeneous receptors that significantly improve the performance of the optimum homogenous ensemble since they are already highly uncorrelated. On the other hand, for the theoretical model the best performance by homogenous ensembles is made with relatively correlated receptor types and therefore, when mixing less correlated receptors (RR = 10–15% and RR = 50–55%), the final ensemble performs significantly better.

### Receptor Distribution in the OB

**Figure 8:**
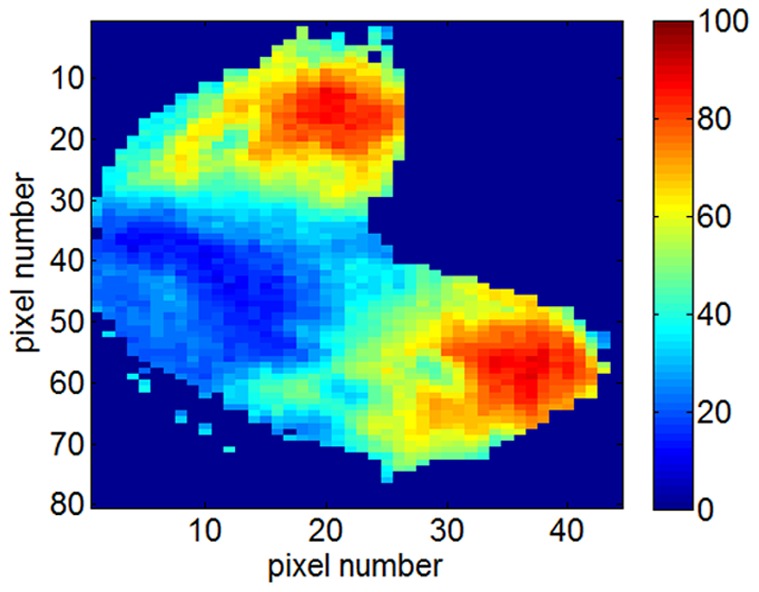
Receptive range of the rat olfactory receptors measured across the olfactory bulb. Less selective receptors are grouped in the medial-caudal and lateral-caudal parts of the olfactory bulb while selective receptors are located in the ventral region.

Figure 8 shows the RR of the measured rat olfactory receptors across the olfactory bulb. We can conclude that the less selective receptors are grouped in the medial-caudal and lateral-caudal parts of the olfactory bulb. Hence, these regions of the olfactory bulb show significant response to most of the odorants and the selective receptors - which surprisingly are significantly correlated (see Figure 7) - are located in the ventral region.

## Discussion

We explored the odor coding capabilities of biological systems using measured activity maps in the rat olfactory bulb across a large set of odorants. From this study, we can infer a number of lessons learned that may help when designing or even analyzing the performance of Artificial Olfaction Systems as general purpose gas and volatile sensing systems.

We estimated the RR of the receptors as the ratio between the number of odorants at which the receptor shows a positive response and the total number of odorants. We encountered a wide diversity of RR, from 10% to 90%, and we found that less selective types of receptors (high RR) are grouped in the medial-caudal and lateral-caudal of the olfactory bulb whereas more selective receptors are in the ventral region. These results are in agreement with experimental studies that show the non-specificity and the RR diversity of the odorant receptors [17] [20]–[23] [25].

In this study we used a public database where the activity in the glomerular layer of the rat OB was mapped using uptake of radiolabeled 2DG. The activity was recorded for a large set of odorants with different chemical properties. We assumed that each pixel corresponds to a single glomerulus and that the same pixels across different images correspond to the same glomerulus. Both approximations are necessary to compare the activity of different stimuli. However, statistical techniques applied to different individuals and stimuli validated these approximations [37].

Due to the limitations of the experimental technique used to acquire the activity map in the rat OB, our study does not consider the information contained in the temporal dynamics of the ORN. Recently, Raman et al. [28] presented a model of the insect antennal lobe to show that odorants are coded as spatiotemporal maps. However, the temporal significance of the ORN responses to code different stimuli has not been fully elucidated yet. Moreover, the contribution to the temporal pattern is twofold: on the one hand it is evoked internally by the temporal dynamics of the neurons, and on the other hand it is driven by the active sensing behavior and fluid dynamics [41]. Therefore, for a better understanding of temporal coding, new databases must be completed defining the temporal signal using microstimulation before system stimulation [42].

In our study we normalized the activity maps to eliminate the dependence on the odor concentration since we are interested in the odor quality recognition regardless of its concentration. Separate studies showed that the activity patterns evoked were constant for odors with the same odor quality in humans after z-score normalization [38] and the normalization keeps the information on odor quality constant despite odor concentration [43].

Therefore, we compared our results with a theoretical model presented by Alkasab et al [30], which only considers the spatial activation in the OB. Alkasab receptors are binary detectors, being independent of the odor concentration. Finally, Alkasab’s model assumes equal probability for the input stimuli (odorants). Despite the very simple theoretical model presented by Alkasab et al. [30], they found that there is an optimum RR when the number of receptors is finite. That is to say, very selective or very unselective receptors performed poorly compared to medium RR receptors. Additionally, in their model, arrays containing different sized receptors perform better than uniform arrays. We found that the odor coding capacity of ensembles of olfactory receptors with the same RR is maximized when RR = 50%. However, the ensemble performance increases only slightly when mixing receptors of different RR. The increase in the performance when using heterogeneous ensembles is smaller compared to the theoretical model due to the low correlation between olfactory receptors. Finally, we found that adding more receptors to the ensemble increases the odor coding performance.

This study has paid special attention to the role played by the receptive range in chemical information encoding. As mentioned before, an RR of 50% seems to be optimal to create systems with a finite number of receptors. It is important for the receptor set to have a good coverage of the odor space defined by the collection of odorants of interest. This can be observed by the fact that all odorants excite more than one pixel, giving redundancy and resilience to receptor failure. However, biological systems do not show homogeneous receptive ranges, but a large variety of them from quite selective receptors to very broadly-tuned receptors. There could be evolutionary reasons for this diversity, since the detection of the different analytes probably does not have the same biological relevance. One may easily envision that biology has evolved toward a combination of more selective sensors for critical odorants and a collection of less selective sensors to cover maximum areas of the odorant space. An extreme case of this evolutionary drive is the presence of highly specific sensors for pheromone detection.

We would like to highlight, however, that there are additional considerations. An additional look at the odorant/receptor Cartesian matrix is provided by the correlation coefficient. Biological sensors show a remarkably low correlation except for very broad receptive ranges. Surprisingly, the analysis of these sets of images also gives a non-negligible correlation for low RR receptors. A deeper study is needed to understand if this correlation is an experimental artifact or whether it has a deeper meaning.

Separate studies [44] have shown that MOX sensors are highly correlated. This is a major lesson for designing sensor arrays. In fact, the advantages of heterogeneous sensor arrays were recognized long ago in the sensor literature [45]. However, since they are more expensive due to the complexity of the hardware, on many occasions homogeneous ensembles of sensors in terms of sensor technology are preferred.

Sometimes we claim that chemical sensors are not selective enough. However, the present study shows that selectivity may not be the most relevant parameter. While the biological system shows a large degree of diversity in RR (in the main olfactory system), we have demonstrated that the optimal performance corresponds to a set of sensors with 50% RR, so they are not particularly selective. Nevertheless, the biological system has a remarkably low correlation and good coverage of the odor input space. For low correlated sensors, adding sensors to the ensemble maximizes the coding capacity of the system.
